# Male breast cancer: a report of 127 cases at a Moroccan institution

**DOI:** 10.1186/1756-0500-4-219

**Published:** 2011-06-29

**Authors:** Mouna Bourhafour, Rhizlane Belbaraka, Amine Souadka, Hind M'rabti, Fouad Tijami, Hassan Errihani

**Affiliations:** 1 Department of Medical Oncology, National Institute of Oncology, Rabat-10000, Morocco; 2 Department of Surgical Oncology, National Institute of Oncology, Rabat-10000, Morocco

## Abstract

**Background:**

Male breast cancer (MBC) is a rare disease representing less than 1% of all malignancies in men and only 1% of all incident breast cancers. Our study details clinico-pathological features, treatments and prognostic factors in a large Moroccan cohort.

**Findings:**

One hundred and twenty-seven patients were collected from 1985 to 2007 at the National Institute of Oncology in Rabat, Morocco.

Median age was 62 years and median time for consultation 28 months. The main clinical complaint was a mass beneath the areola in 93, 5% of the cases. Most patients have an advanced disease. Ninety-one percent of tumors were ductal carcinomas.

Management consisted especially of radical mastectomy; followed by adjuvant radiotherapy and hormonal therapy with or without chemotherapy. The median of follow-up was 30 months. The evolution has been characterized by local recurrence; in twenty two cases (17% of all patients). Metastasis occurred in 41 cases (32% of all patients). The site of metastasis was the bone in twenty cases; lung in twelve cases; liver in seven case; liver and skin in one case and pleura and skin in one case.

**Conclusion:**

Male breast cancer has many similarities to breast cancer in women, but there are distinct features that should be appreciated. Future research for better understanding of this disease at national or international level are needed to improve the management and prognosis of male patients.

## Background

Male breast cancer is a rare disease that accounts for less than 1% of all cancers in men and less than 1% of all diagnosed breast cancers [1]. The literature regarding male breast cancer consists mainly of retrospective studies, and there are no randomized prospective data for this disease. This is an entity for which optimum management and treatment guidelines are not clearly established. Generally, treatment recommendations have been extrapolated from results of trials in female patients.

In this study, we retrospectively evaluated the clinico-pathological features, treatments and the results obtained in 127 cases of male breast cancer treated at the National Institute of Oncology in Rabat, Morocco, between 1985 and 2007.

## Patients and methods

### Clinical data

The investigation was a retrospective (the data was collected by chart review), observational, single-centre study.

Inclusion criteria were: male patients > 18 years old with localized, locally Advanced or metastatic breast cancer.

We excluded from the study patients who had not follow up after initial diagnosis.

Breast carcinoma diagnosis was made by biopsy of the breast tumor. Tumor staging was carried out according to the TNM classification 2002 modified in 2003. Histological tumor grading was performed using the Scarff Bloom and Richardson (SBR) histological system.

Immunohistochemical analysis to determine estrogen (ER) and progesterone receptor (PR) status was performed using standard procedures on 4-μm sections of paraffinembedded tissue specimens stained with the monoclonal antibodies 6F11 and 1A6 for ER and PR, respectively.

Nuclear staining 10% was considered a positive result.

### Statistical analysis

Descriptive of clinical data were expressed in percentage or median or mean ± SD. Survival was estimated by the Kaplan Meier method, and compared by the log rank test. The relationship between each of the explanatory variables and outcome (EFS and OS) was assessed in turn using univariate and multivariate Cox's regression analysis. A p value of < 0.05 was considered significant.

### Consent and statement of ethical approval

As the treatment of each patient was decided by the medical staff of the centre, oral consent was obtained from the subjects and was approved by the institutional review boards of the National Institute of Oncology, Cancer Centre in Rabat. This study was approved by the institutional review boards of National Institute of Oncology, in Rabat.

## Results

### Clinical characteristics

One hundred and twenty-seven patients at the National Institute of Oncology in Rabat, Morocco with a diagnosis of breast cancer between January 1985 and December 2007 were analyzed retrospectively and evaluated in terms of general characteristics and survival.

The median age was 62 years (range 32-91 years). A family history of breast cancer was noted in four cases.

The main clinical complaint was a mass beneath the areola in 93, 5% of the cases. The tumor was associated with gynecomastia in 4% of cases. Paget's disease was found in 2, 5% of cases. The median time for consultation was 28 months (range: 3-48 months).

According to the TNM classification, tumors were categorized as T1: 6 cases (5%), T2: 18 (14%), T4 and T3 in, respectively, 62, 5% and 17% of the cases and Tx (unclassified tumors): 2 cases (1, 5%). Tumors were classed as N1 and N2, respectively, in 55, 2% and 25, 2% of the cases. Thirty seven patients (29%) initially had metastases. Table 1 resumes clinico-pathological features.

**Table 1:** Clinico-pathological features

	0- 49	20 cases	16%
	50-59	35	27,5%
Age (years)	60-69	50	39%
	> 70	22	17,5%
	Mass beneath the areola	119 cases	93,5%
Clinical complaint	Gynecomastia	5	4%
	Paget'disease	3	2,5%

	Tx	2 cases	1,5%
	T1	6	5%
Primary Tumor	T2	18	14%
	T3	22	17%
	T4	79	62,5%

	Nx	9	7,1%
Lymph node	N0	16	12,5%
	N1	70	55,2%
	N2	32	25,2%

Metastasis	M0	90	71%
	M1	37	29%

	I	6	5%
	IIA	8	6,1
Stage	IIB	12	9,6
	III	64	50,3%
	IV	37	29%

	IDC:	122	96%
	SBR 2 ou 3	100	82%
Histology	With Paget's disaese	2	1,6%
	ILC	1	0,8%
	ND	2	1,6%

Lymph node status	pN0	29	35,5%
	pN+	54	64,5%

Hormone receptors	RE	39/61	64%
	RP	39/61	64%

Ductal infiltrating carcinoma (IDC); Invasive lobular carcinoma (ILC); not defined (ND) Estrogen receptor (ER); progesterone receptor (PR)

Ductal infiltrating carcinoma (IDC) corresponded to 122 cases (96%), infiltrating ductal carcinoma with Paget's disease of the nipple in two cases and Invasive lobular carcinoma (ILC) in one case.

According to the Scarff-Bloom-Richardson grading, grade II or III was predominant (82% of the cases). Axillary lymph nodes contained metastasis (N+) in 64, 5% of the cases (54 patients).

Moreover, hormone receptors were evaluable in sixty one cases. Both Estrogen receptor (ER) and progesterone receptor (PR) were positive in 64% (39 patients).

### Treatment

The treatment consisted of radical mastectomy (RM) in 71% (90 cases); modified radical mastectomy (MRM) in seven cases; total mastectomy without axillary node dissection (AD) in seven cases and lumpectomy (L) in one case.

All patients received adjuvant therapy following surgery. Sixty patients of ninty patients received radiotherapy; the median delivered doses were 50 Gy to breast, chest wall and regional lymph nodes. Chemotherapy (an anthracycline-based protocol; AC60 or FEC 100) was given in neoadjuvant situation in five cases, in adjuvant situation in 23 cases. Chemotherapy delivery increased according particularly to advanced stage and axillary nodal involvement. Nine cases receive it in palliative situation.

Hormonal therapy was delivered to fifty-seven patients as adjuvant situation: Tamoxifen alone in 50 cases, Tamoxifen with orchidectomy in four cases and castration in three cases. Seventeen patients received it in palliative situation. Table 2 summarize the treatment modalities according to the TNM stage

**Table 2:** Treatment modalities according to the TNM stage

	Surgery	6 cases	
Stage I (nb = 6)	Adjuvant		5 total mastectomy without AD, 1 (L)
	Treatment		0
	Surgery	8 cases	
Stage II A (nb = 8)			2 total mastectomy without AD, 6 MRM
	Chemotherapy		0
	Radiation therapy		0
	Hormonal therapy		0

	Surgery	12 cases	
Stage IIB (n = 12)			1 MRM, 11 RM
	Chemotherapy		3
	Radiation therapy		8
	Hormonal therapy		6

	Surgery	64 cases	
Stage III (n = 64)			59 RM immediatly
			5 RM after neoadj CMT
	neoadjuvant CMT		5
	adjuvant CMT		20
	adjuvant RTH		52
	adjuvant HT		51

	Surgery	15 cases	
Stage IV (n = 37)			15 RM
	CMT		9
	RTH		9
			10(bone'metastasis)
	HT		17
	BSC		11

Lumpectomy (L); modified radical mastectomy (MRM); radical mastectomy (RM); axillary dissection (AD); Chemotherapy (CMT); Radiation therapy (RT); Hormonal therapy (HT); Best supportive care (BSC);

During the median follow-up period of 30 months (3-168 months); the evolution has been characterized by local recurrence in twenty two cases (17% of all patients). Metastasis occurred in 41 cases (32% of all patients). The site of metastasis was the bone in twenty cases; lung in twelve cases; liver in seven case; liver and skin in one case and pleura and skin in one case.

**Figure 1:**
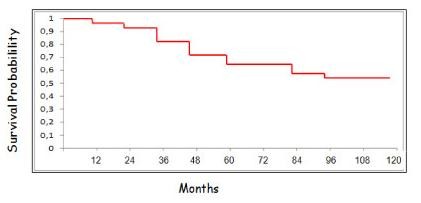
**Overall Survival**.

The 5- and 10-year overall survival (OS) rates were 63% and 55%. (Figure 1)

## Discussion

Carcinoma of the male breast has many similarities to breast cancer in women, but there are distinct features that should be appreciated. During the last few years, there has been an increase in the incidence of this disease. Review of Surveillance, Epidemiology and End Result (SEER) data indicate a rise in the incidence of male breast cancer, from 1.0 per 100,000 men in the late 1970s to 1.2 per 100,000 men from 2000 to 2004 [1,2].

The mean age at diagnosis for men with breast cancer is 67 years, which is approximately 5-10 years older than the average age at diagnosis for women [1,3]. The mean age in our patients (62 years) is lower than other series.

Male breast cancer is likely to be caused by the concurrent effects of different risk factors, including clinical disorders relating to hormonal imbalances, certain occupational and environmental exposures, and genetic risk factors, for instance a positive family history of breast cancer and mutations in breast cancer predisposing genes, such as *BRCA *genes, and possibly others [3].

The most common clinical sign of breast cancer onset in men is a painless palpable retroareolar lump [4]. Other initial symptoms may include nipple involvement, with retraction and/or ulceration and/or bleeding, axillary lymphoadenopathies and gynecomastia [3,4].

As male breast does not have lobular elements, the most frequently encountered male breast cancer type is invasive ductal carcinoma (IDC) (85-95%) [4,5]. The result in our study was similar with a ratio of 96% for IDC and this was significantly higher than the other histological types. Lobular carcinoma in situ, Paget disease and inflammatory breast cancer have been rarely described in men[5].

Positivity rate of receptors is more frequent in men with breast cancer, in comparison to women [6]. In different studies, ER and PR positivity was reported as 75 to 93% [7,8]. In our study, both Estrogen receptor (ER) and progesterone receptor (PR) were positive in 64%.

Breast cancer in men should be treated with the same strategy as in women [9,10]. The most common surgical procedure is modified radical mastectomy with axillary node dissection [11]. However, recent studies are in favor of modified radical or simple mastectomy combined with radiation therapy. Postoperative radiotherapy does achieve local control but no effect is observed on survival [12]. In men treated with mastectomy, adjuvant radiotherapy has shown to decrease local recurrence [13].

Tamoxifen has proved to lead to an increase in survival rates in women with hormone-responsive disease and to date is generally considered the standard adjuvant treatment for hormone-dependent male breast cancer [14]. The tolerance of the drug has not been sufficiently studied in men; its main side effects are deep venous thrombosis, reduction of libido, impotence, mood changes and hot flushes [15].

Chemotherapy should be used in the absence or doubt about endocrine-responsiveness. Frequently used chemotherapy regimens were CMF, FEC and EC [16]. The taxanes may be considered when lymph nodes are involved. Regarding the use of adjuvant trastuzumab, since no specific data exist, its use should be considered according to patients' and tumor characteristics [17,18].

The overall 5- and 10-year survival rate of male breast cancer patients are around 60 and 40%, respectively [17]. The number of histologically positive axillary nodes and the tumor size are significant prognostic factors. Another negative prognostic factor is the advanced age at the time of diagnosis, since the increased presence of comorbidities may limit the possibility of treatment [18].

## Conclusion

Lifetime in men with breast cancer is worse than in women. While some investigators explain this with more aggressive biologic behavior of male breast cancer, more frequent explanation is the rareness of male breast cancer and achieving its diagnosis at a more advanced stage.

Without a concerted effort, the literature pertaining to male breast cancer will remain a collection of retrospective series and pilot studies. Efforts to develop randomized, prospective studies within cooperative groups and other clinical trial consortia are essential.

## Abbreviations

MBC: Male breast cancer; SBR: Scarff Bloom and Richardson; ER: Estrogen receptor; PR: progesterone receptor; IDC: Ductal infiltrating carcinoma; ILC: Invasive lobular carcinoma; RM: Radical mastectomy; MRM: Modified radical mastectomy; AD: Axillary node dissection; L: Lumpectomy; AC60: doxorubicin 60 mg/m^2 ^and cyclophosphamide 600 mg/m^2^; FEC100: 5-fluorouracile 500 mg/m^2^, epirubicin 100 mg/m^2^, and cyclophosphamide 500 mg/m^2^; EFS: event free survival; OS: overall survival.

## Competing interests

The authors declare that they have no competing interests.

## Authors' contributions

MB and RB: drafted the manuscript. RB: participated in the design of the study and review of the final manuscript and revising it critically for important intellectual content.

All authors read and approved the final manuscript.
